# Knowledge, attitude and practice of Iranian hypertensive patients regarding hypertension

**DOI:** 10.15171/jcvtr.2018.02

**Published:** 2018-03-17

**Authors:** Yaseen Rashidi, Hesam Manaflouyan, Fatemeh Pournaghi Azar, Zeinab Nikniaz, Leila Nikniaz, Samad Ghaffari

**Affiliations:** ^1^Student Research Committee, Tabriz University of Medical Sciences, Tabriz, Iran; ^2^Iranian Center for Evidence-Based Medicine, Tabriz University of Medical Sciences, Tabriz, Iran; ^3^Liver and Gastrointestinal Disease Research Center, Tabriz University of Medical Sciences, Tabriz, Iran; ^4^Tabriz Health Services Management Research Center, Tabriz University of Medical Sciences, Tabriz, Iran; ^5^Cardiovascular Research Center, Tabriz University of Medical Sciences, Tabriz, Iran

**Keywords:** Hypertension, Knowledge, Attitude, Practice, Iran

## Abstract

***Introduction:*** This study aimed at evaluating knowledge and awareness of hypertension and the risk factors for hypertension among hypertensive patients.

***Methods:*** In this study, 110 hypertensive patients were enrolled and filled out two self-administered questionnaires. The first questionnaire was about the demographic characteristics and the second one was about the knowledge (n = 10), attitude (n = 9) and practice (n = 8). The internal consistency and the stability of the questionnaires were approved. The Mann-Whitney U test and Kruskal-Wallis and Spearman correlation coefficient were used for statistical analysis.

***Results:*** Seventy-three percent of participants know the normal range of hypertension. Most of the participants truly knew that stress (87.3%), obesity (70.9%) and aging (48.2%) are risk factors for hypertension. About 60% of participants knew the complications of uncontrolled hypertension. About 82.7% of participants believed that after adaptation of body to hypertension, there is no need to use antihypertensive drug. About 13.6% of participants measured their blood pressure daily and 11.8% of them measured it once a month. The educational level of participants was significantly associated with knowledge score (*P * = 0.01). There was a significant correlation between knowledge (*P * < 0.001) and attitude and also attitude and practice (*P * < 0.001) scores.

***Conclusion:*** These findings have important implications for developing proper and continuous self-management hypertension education programs in Iran which should mostly emphasize on the practical information about control and prevention programs.

## Introduction

Hypertension is a major risk factor for chronic diseases and deaths worldwide with age-standardized prevalence of 24.1% and 20.1% in men and women respectively.^1^ This number is growing very fast and it is estimated that the number will reach to more than 1.56 billion by the year 2025.^2^ Hypertension is also responsible for 57 million disability adjusted life years (DALYs) and it is estimated that about 7.5 million deaths (12.8 of all-cause deaths) worldwide is due to high blood pressure.^3^

In spite of a high prevalence, the ratio of taking blood pressure under control among hypertensive patients is still very low. According to Kilic et al, this value is only 30%-34% in developing and 33%-38% in developed countries.^4^

Most hypertensive people are not aware of their condition or have a low level of health literacy. Having an inadequate level of knowledge about the health issues has been reported for the hypertensive patients in different countries all over the world such as the United States,^5^ Pakistan,^6^ Turkey,^4^ and Namibia.^7^ In a study carried out in sub-Saharan Africa communities, Hendriks et al showed that only 3% of hypertensive people in Namibia were aware of their condition; this value was found to be 6% for the patients in Kenya.^7^

The knowledge, perceptions and attitude of people towards hypertension has a significant role in changing lifestyle including the modifiable risk factors of hypertension. It has been shown that self-management behaviors such as taking prescribed medications, quit smoking, eating a healthy diet and increasing physical activity level are crucial for hypertensive patients. Therefore, it would be possible to reduce burden of hypertension by changing the modifiable risk factors through increasing the health literacy of hypertensive patients.^8^ Finland is a good example here. In an awareness campaign concerning cardiovascular diseases launched in 1974, the mortality rate was reduced to more than half just in 25 years.^9^

Like most countries, high blood pressure is one of the major causes of deaths in Iran.^10^ According to Malekzadeh et al, of the total cohort participants in northeastern of Iran, 43% were hypertensive.^11^ Despite this high prevalence, studies examining awareness of hypertension in Iran is scarce. The information in these issues will help to develop suitable intervention programs aimed at increasing disease self-management behaviors among hypertensive patients. To fill this crucial gap, in the present study, knowledge and awareness of hypertension and the risk factors for hypertension among hypertensive patients have been investigated.

## Materials and Methods

In the present cross-sectional study, 110 hypertensive patients who referred to Shahid Madani hospital were participated. Males and females subjects who had diastolic blood pressure >140 and systolic blood pressure >90 on two consecutive reading and aged >30 were participated in the present study.

Participants completed two self-administered questionnaires. The first questionnaire was about the demographic characteristics and the second one was about the knowledge (n = 10), attitude (n = 9) and practice (n = 8).

### Item extraction

In order to develop the questionnaire, an extensive literature review was undertaken. Items which seemed appropriate for our questionnaire were extracted and a primary questionnaire was designed. For content validity of the questionnaire, the primary questionnaire reviewed by a panel of 10 experts. The 4 point Likert scale which assessing the relevance, clarity, simplicity and necessity of the primary questionnaire was filled in by experts content validity index (CVI) and content validity ratio (CVR), were calculated and questions with CVI <0.79 and CVR <0.69 were excluded.^12,13^

The internal consistency of the instrument was approved using Cronbach α (0.70) in a pilot study of 10 patients. In the same patients after two weeks, the test-retest was performed for assessing the stability of the questionnaire. The Spearman–Brown index was 0.75 and the intra-class correlation coefﬁcient was 0.72, 95% CI (0.46, 0.83).

### Statistical analysis

SPSS version 18 statistical computer software was used for all statistical analyses. Normal distribution was assessed using Kolmogorov-Smirnov test. Each multiple-choice question had one correct answer that was assigned the score of 1 point; whereas, 0 point was assigned to all wrong answers. Mann-Whitney U test and Kruskal-Wallis were conducted to compare the median of correct responses of every section by gender, age, educational level and duration of the disease. For the cor­relates analyses, Spearman correlation coefficient was used for investigating the association between knowledge, attitude and practice (KAP) scores. The significance level of .05 was used.

## Results

Table 1 outlines the demographic characteristics of participants. Totally, 110 participant including 52 males and 58 females with the mean age of 57.97±10.67 years were participated in the present study. About 95.5% of participants were married and lived in rural areas. Among the participants 11.8% of them were illiterate and 2.7% were unemployed.

**Table 1:** Demographic characteristics of the participants

**Variables**	
Age (y), Mean ± SD	57.97 ± 10.67
Duration of the disease (y), Mean ± SD	8.30 ± 6.75
Sex, No. (%)	
Male	52 (47.3)
Female	58 (52.7)
Marital status, No. (%)	
Married	105 (95.5)
Single	5 (4.5)
Residency, No. (%)	
Urban	5 (4.5)
Rural	105 (95.5)
Education, No. (%)	
Illiterate	13 (11.8)
≤High school/diploma	49 (44.5)
≥ College degree	48 (43.6)
Employment status, No. (%)	
Employed or self employed	34 (30.90)
Unemployed	3 (2.7)
Homemaker	40 (36.4)

The median knowledge (total score 12), attitude (total score 9) and practice (total score 10) scores of participants is summarize in Table 2. The median (IQR) score of knowledge, attitude and practice of participants were 7 (2), 7 (3) and 4 (2) respectively.

**Table 2:** The median of knowledge, attitude and practice scores

	**Median (IQR)**	**Min. score**	**Max. score**
Knowledge (max score 12)	7 (2)	3	10
Attitudes (max score 9)	7 (3)	2	9
Practice (max score 8)	4 (2)	1	7

Table 3 shows the percent of correct answer for each question. According to this table 72.7% of participants know the normal range of hypertension but only 30.9% of them know that hypertension usually have no sign. Most of the participants were truly know that stress (87.3%), obesity (70.9%) and aging (48.2%) are risk factors for hypertension. On the other hand about 85.5% of participants say that toxins are risk factors for hypertension. About 60% of participants know the complications of uncontrolled hypertension. The majority of participants think that increasing vegetable consumption (91.85) and also regular physical activity (84.5%) could prevent hypertension but in practice only 37.27% of them increase their physical activity. About 82.7% of participants believed that after adaptation of body to hypertension, there is no need to use antihypertensive drug. About 13.6% of participants measured their blood pressure daily and 11.8% of them measured it once a month. About 76.36% of participants reduced their salt intake and 59.09% of them reduced their fat intake. About 25.5% of participants use alternative medicine in addition to using antihypertensive drugs.

**Table 3:** Correct responses to foods that increase risk of food borne disease scale questions

**Question**	**No. (%) of Correct Response**
**Knowledge**	
1. Do you know the normal BP reading?	80 (72.7)
2. Hypertension usually have no sign	34 (30.9)
3. Is stress a risk factor for hypertension	96 (87.3)
4. Is obesity a risk factor for hypertension	78 (70.9)
5. Is toxins a risk factor for hypertension	94 (85.5)
6. Is aging a risk factor for obesity	53 (48.2)
7. Which age group is more susceptible for high blood pressure?	12 (10.9)
8. If hypertension s not controlled, which of these complications could raised?	66 (60)
9. The prevalence of hypertension in subjects <30 years is low	12 (10.9)
10. There's no identifiable cause of high blood pressure	31 (28.2)
11. Are Analgesic drugs one of the risk factors for high blood pressure?	35 (31.8)
12. Is blood pressure hereditable?	68 (61.8)
**Attitude**	
1. Should we increase vegetable intake to prevent hypertension?	101 (91.8)
2. Should we have regular physical activity to prevent hypertension?	93 (84.5)
3. Do you think that antihypertensive drugs should only used under stress situations?	43 (39.1)
4. Do you think that for hypertension control, the patient should use antihypertensive drug all over life	92 (83.6)
5. Should Hypertensive patient use anti hypertension drug before exercise	38 (34.5)
6. Do you t6hink that the prevalence of hypertension in young people is very low	77 (70)
7. For hypertension diagnosis, the blood pressure should be measured and only the sign are not enough	78 (70.9)
8. After adaptation for hypertension, there is o need to take drugs any more	91 (82.7)
9. Hypertension is dangerous but controllable disease	107 (97.3)
**Practice**		
1. How often do you measure your blood pressure?	
Daily	15 (13.6)
Twice a week	20 (18.2)
Once a week	15 (13.6)
Monthly	13 (11.8)
If I have a problem	47 (42.7)
2. Where do you measure your blood pressure?
Physician office	29 (26.4)
Health centers	30 (27.31)
At home through family members	89 (80.9)
At home through nurses	1 (0.9)
3. Do you used your anti hypertensive drugs according to physician order?
Yes	104 (94.5)
4. Do you reduce your salt intake?	84 (76.36)
5. Do you reduce your fat intake?	65 (59.09)
6. Do you have regular physical activity?	41 (37.27)
7. What are you doing if you experience the side effects of antihypertensive drugs?
Drug withdrawal	9 (8.2)
Consulted with another physician	7 (6.4)
Consulted with your physician	94 (85.5)
Visit hospital	9 (8.2)
8. Do you use alternative medicine for hypertension in addition to antihypertensive drugs?
Yes	28 (25.5)

Table 4 shows the differences in KAP scores across different demographic variables. The statistical analysis showed that only the educational level of participants was significantly associated with knowledge score (*P* = 0.01).

**Table 4:** Differences in knowledge and attitude scores across different demographic variables

**Variables**		**Knowledge**		**Attitude**		**Practice**	
		**Score**	***P *** **value**	**Score**	***P *** **value**	**Score**	***P *** **value**
Sex	Male	7 (2)	0.28*	6 (2)	0.10	4 (2)	0.29
	Female	7 (2)		7 (3)		4 (1)	
Education level	Illiterate	6 (3.5)	0.01**	7 (3)	0.23	4.5 (3)	0.84**
	≤High school/diploma	6 (2)		7 (3)		5 (1.75)	
	≥ College degree	7 (2)		7 (2)		4 (1)	
Duration of hypertension	1-2 years	7 (1.5)	0.79**	7 (3)	0.11	4 (1)	0.35**
	3-4 years	6 (3)		7 (3)		4 (2.5)	
	>5 years	7 (2)		7 (3)		4 (1.75)	

*P value Man-whitney U. **P value Kruskal-Wallis.

Table 5 depicted the correlation coefficient between knowledge-attitude-practice scores and the results showed that there was a significant correlation between knowledge (*P* < 0.001) and attitude and also attitude and practice (*P* < 0.001) scores.

**Table 5:** Correlation between knowledge, attitude and practice scores

**Variables**	**Correlation coefficient**	***P*** value*
Knowledge-attitude	0.30	0.001
Knowledge-practice	0.10	0.29
Attitude-practice	0.35	<0.001

*P value Pearson correlation coefficient.

## Discussion

It is of great importance for hypertensive patients to discover the KAP level of them to develop the appropriate educational and self-management programs. In this regard in the current cross-sectional study the KAP of hypertensive patients in Tabriz-Iran was studied. According to results, the overall scores of participants were medium except for attitude that is higher than other variables. In another studies from Iran, it has been reported that in more than 50% of the participants, the knowledge level of participants regarding hypertension was average.^14,15^ It seems that comparing with other studies conducted in Nepal,^16^ the United States^17^ and Mongolia,^18^ the present study reported lower scores on the issue. The difference between studies could result from differences in the educational level of participants, using different tools for assessing the KAP level and availability of educational programs in different countries.

Majority of the hypertensive patients in the present study had a high knowledge regarding the obesity and stress as risk factors of hypertension. The link between obesity and stress and hypertension has been the subject of various review articles^19^ and it has been shown that this may be due to activation of the sympathetic nervous system, renin-angiotensin system, and also sodium retention.^19^ Along with their knowledge regarding obesity their attitudes toward changing their diet and increase their physical activity was also high. Although their practice regarding the lowering consumption of salt and also high fat foods was good, the practice of increasing the physical activity level was not impressive and they did not put their knowledge into practice. The differences between knowledge and practice may be due to the this fact that they know that for controlling high blood pressure, they should reduce their weight by diet and also increasing physical activity but they may not have enough knowledge regarding the appropriate ways to do this.

Previous studies in Pakistan^6^ and Nepal^16^ showed that there was a significant difference in the KAP level of males and females. However, in another study in Nepal, there was no association between KAP level and sex. In the present study we also did not observe the significant differences in KAP scores between males and females but the educational level was significantly associated with knowledge scores.^20^ The plausible explanation of these results is that there were not significant differences in the educational level of males and females in the present study. Previously, it has been shown that the literate people had better knowledge than illiterate ones.^16^ Consistent with the results of study conducted in Nepal, there was not significant association between disease duration and KAP scores.^20^ So, it seems that education intervention in any stage of the disease could have significant influence on patients practice.

About 25% of our participants use complementary alternative medicine (CAM). The use of CAM in present study was similar to the studies done in Nigeria 29%,^21^ Ghana 19.5%^22^ and South Africa 21%^23^ but it is lower than the studies done India (63.9%).^24^ The differences in the prevalence of CAM use across different countries may be due to the variations in sociocultural background and accessibility of modern medical practice.^25^ As most of the information about CAM were suggested by families, relatives, and friends (49%), to prevent the side effects of CAM use, it is important to inform health care practitioner about the type and amount of CAM and get proper information from medical centers.^26^

One of the important limitations of the present study is low sample size. Also, the self-reported practice which is prone to bias by participants is the other important limitation. So, the results should be interpreted considering these limitations.

## Conclusion

The prevalence of hypertension in Iran is increasing and for decreasing the burden of disease, focusing on KAP of patient by implementing the educational intervention is essential. Accordingly, the results of the present study showed that the knowledge and practice of hypertensive patients is medium. These findings have important implications for developing proper and continuous self-management hypertension education programs in Iran which should mostly emphasize on the practical information about the popper interval of checking blood pressure, signs of the disease, proper and practical methods of increasing physical activity. Furthermore, despite the good knowledge of some respondents, their food safety practices were poor. Therefore, the educational programs should be repeated with proper intervals to ensure that learnt information is turned into practice. Moreover appropriate self-management programs could also designed to improve the living condition of these patients and reduce costs for patients and also health system. According to results of the present study, individuals with lower educational level may benefit more from educational programs. Additionally, as there is significant association between knowledge and practice in hypertensive patients, for all patients the educational programs should be practice oriented.

## Ethical approval

The ethical approval for this study was obtained from Ethics Committee of Tabriz University of Medical Sciences.

## Competing interests

The authors declare no conflict of interest.
